# Targeting mutant p53 for cancer therapy

**DOI:** 10.18632/aging.100992

**Published:** 2016-06-26

**Authors:** Moshe Oren, Perry Tal, Varda Rotter

**Affiliations:** Department of Molecular Cell Biology, The Weizmann Institute, Rehovot 76100, Israel

**Keywords:** TP53, mutant p53 gain-of-function, peptide therapy, tumor suppressor, MDM2

The p53 tumor suppressor protein serves as a major barrier against cancer; consequently, mutations in the TP53 gene, encoding p53, are the most frequent single genetic alteration in human cancer, occurring in about half of all individual cancer cases [1]. Besides abrogating the tumor suppressive effects of the wild type (WT) p53 protein, many of the TP53 mutations endow the mutant p53 protein with new oncogenic gain-of-function activities, which actively promote a variety of features characteristic of aggressive tumors, such as increased migratory and invasive capacities and increased resistance to many types of anti-cancer therapy agents [1]. This pertains particularly to tumors that carry single amino acid substitutions (missense mutations) within p53's DNA binding domain (DBD), and display abundant accumulation of the mutant p53 protein within the tumor cells [1].

In tumors that retain non-mutated TP53 genes, the tumor suppressive effects of the remaining WTp53 are also often compromised, owing to genetic and epigenetic alterations that occur during cancer progression [1]. Altogether, the normal functionality of p53 is thus abrogated in the vast majority of human tumors. This realization has led to extensive attempts to restore full p53 functionality in cancer cells, as a novel cancer therapy strategy [1, 2]. However, these attempts have been seriously hampered by the fact that p53 has no known enzymatic activities, and rather operates primarily as a sequence-specific transcription factor. Furthermore, restoring the activity of a defective tumor suppressor protein is vastly more difficult than abrogating the activity of a hyperactive oncoprotein.

Nevertheless, significant advances have been achieved in recent years, and hopes for the introduction of p53-based novel cancer therapies into the clinic are becoming increasingly supported by evidence. In principle, attempts to develop such therapies have taken 3 main approaches: (1) Introduction of WTp53, mainly via viral transduction (“gene therapy”), into tumors that have sustained TP53 mutations; (2) enhancement of the functionality of the endogenous WTp53 in tumors that have retained a non-mutated TP53 gene, mainly be disrupting the interaction of the WTp53 protein with its major negative regulator MDM2; and (3) “correction” of the mutant p53 protein in tumors that have sustained TP53 missense mutations, thereby restoring its ability to perform the tumor suppressive activities of WTp53 [1, 2].

The latter approach, namely the “re-education” of mutant p53, is particularly appealing. First of all, it can simultaneously reinstate WTp53 tumor suppressive activity together with abrogating the gain-of-function oncogenic effects of the mutant p53 protein. Additionally, since cancer cells bearing TP53 missense mutations often accumulate massive amounts of the mutant p53, its conversion into a WT-like state will potentially flood the cancer cell with excessive amounts of tumor suppressive p53, far beyond what one finds in normal cells. This may provide a large therapeutic window and may potentially circumvent the severe limiting toxicity observed with compounds that augment the activity of non-mutated p53 in cancer cells (approach #2 above).

Indeed, attempts to “re-educate” mutant p53 in cancer cells have seen substantial progress in the last several years. The most advanced effort has been spearheaded by Wiman and coworkers, who identified a small molecule named PRIMA-1, which can reactivate mutant p53 (reviewed in [3]). PRIMA-1 was subsequently further modified, and its derivative, PRIMA-1-met, has recently entered a Phase 2 clinical trial under the commercial name APR-246 [3]. An additional strategy, developed by Carpizo, Levine and co-workers (reviewed in [4]), is based on the facts that Zn(2+) ions are crucial for stabilizing the correct folding of the DBD of WTp53, and that many (but not all) cancer-associated mutant p53 proteins bind Zn(2+) less avidly that WTp53 and therefore tend to misfold. Specifically, these investigators have identified small molecules (zinc metallochaperones) that deliver Zn(2+) to the DBD of mutant p53 and facilitate its correct refolding, thereby restoring WTp53-like function [4]. However, such molecules work only on a subset of p53 mutants, which have a conformational defect due to reduced Zn(2+) binding. Moreover, like PRIMA-1/APR-246, they possess a rather generic chemical activity are not specific for p53 only; this may result in undesirable side effects that are presently hard to predict. Recently, El-Deiry and coworkers have described 2 additional mutant p53-targeing small molecules: prodigiosin, which disrupts the interaction of mutant p53 with the p53 family member p73, and thereby unleashes the cytotoxic and cytostatic activities of p73 [5], and NSC59984, which augments p53 degradation and also unleashes p73 activity [6].

We have opted for a different approach, based on identification of small peptides that specifically stabilize mutant p53 proteins in a functional state [7]. Combining phage display screening with several alternating functional readouts, which minimize the frequency of false-positives, we were able to obtain a series of such bioactive peptides. These peptides can stabilize the WT conformation of mutant p53, and restore its ability to engage in sequence-specific DNA binding and activate canonical WTp53 target genes. Moreover, they promote selective apoptotic death of cancer cells harboring mutant p53, and very effectively reduce, and even completely block, the growth of human cell line-derived mouse xenograft tumors representing several types of highly aggressive cancer [7]. Importantly, all common p53 mutants tested in our study were found to be amenable to functional stabilization by these peptides. Remarkably, our lead peptide, pCAP-250, shares perfect homology with the RAD9 protein, a validated p53 interactor. This attests to the high specificity of the interaction.

Of note, Eisenberg and coworkers have recently described another type of mutant p53-targeting peptide, which acts by disrupting the aggregation of particular aggregation-prone p53 mutants [8]. The spectrum of mutants targeted by such peptide still remains to be determined.

Bringing small peptides into the clinic remains challenging, mainly owing to the need to deliver the peptides efficiently into the tumor cells. Nevertheless, their greater specificity, relative to small molecules of the types described above, bears the hope for minimal non-specific toxicity, rendering such approach potentially highly promising in the long run.
